# Greening China’s Growth: Assessing the Synergistic Impact of Financial Development and Technological Innovation on Environmental Pollution Reduction—A Spatial STIRPAT Analysis

**DOI:** 10.3390/ijerph20065120

**Published:** 2023-03-14

**Authors:** Jiachao Peng, Shuke Fu, Da Gao, Jiali Tian

**Affiliations:** 1 School of Law and Business, Wuhan Institute of Technology, Wuhan 430205, China; 2 Center for High Quality Collaborative Development of Resources, Environment and Economy, Wuhan Institute of Technology, Wuhan 430205, China

**Keywords:** synergy effect, financial development, technological innovation, environment pollution, spatial STIRPAT model

## Abstract

To achieve sustainable economic development in China, it is crucial to balance economic growth and environmental protection. Financial capital and technology can contribute positively to environmental pollution control. This study employs the Cournot model to examine the impact of financial development and technological innovation on environmental pollution at the micro level. It utilizes the spatial STIRPAT model to analyze inter-provincial panel data from China between 2005 and 2020. The results show that China’s ecological environment pollution exhibits significant spatial dependence, and heavily polluted areas tend to agglomerate. While improving financial development can increase regional environmental pressure, positive spatial spillover improves environmental quality in neighboring areas. Conversely, technological innovation reduces local ecological pressure, with negative spatial spillover effectively curbing environmental pollution in surrounding regions. The results support the environmental Kuznets curve (EKC) hypothesis, which posits an inverted U-shaped relationship between economic growth and environmental pressure, while population growth increases environmental pressure. The findings are robust and have important policy implications.

## 1. Introduction

The sustainability and progress of human society heavily relies on the preservation of a healthy ecological environment. However, as the global population and production scale continue to expand, total wealth is growing exponentially, and the environment, which is critical for human survival, is facing significant challenges. Recent research by the United Nations Educational, Scientific, and Cultural Organization (UNESCO) has revealed that approximately one-fifth of the world’s population resides in regions with inadequate water supply. The increasing groundwater pollution is aggravating their domestic water usage challenges. The Greenhouse Gas Bulletin, published by the World Meteorological Organization in 2018, highlighted that global average carbon dioxide concentration reached an unprecedented high in 2016, which could result in severe consequences such as a 20 m rise in sea level and a three-degree increase in global temperature. In light of the frequent incidents of environmental pollution in recent years, governments worldwide have become more attentive to environmental protection [1]. To control the increasingly severe environmental pollution problem, governments of various countries have invested a great deal of manpower and material resources, for example, environmental regulation [2,3], pollution-intensive industry transfer [1], and digital technology [4,5]. China is responsible for environmental protection as the world’s most populous country and the second-largest developing economy.

At present, China has begun to enter a new normal of the “three-period superimposed”. Since the Economic Reform and Open up, China’s sustained high economic growth has made it the second-largest economy in the world. However, at the same time, there are huge changes in the environmental resources, natural resources, human capital, and other factors that support economic development at high speed [6]. The continuous haze weather, air pollution, water pollution, soil pollution, ecological crisis, and other environmental degradation in all parts of the country are causing local governments to recognize that the contradiction between economic development and resources and the environment is becoming increasingly acute, and it is important to resolve this contradiction [7]. To achieve sustainable economic development, it is crucial to modify the industrial structure. The transition towards green economic practices requires adequate support from financial institutions and technological advancements. The core of modern society is finance, and the shift towards green economic transformation necessitates the implementation of green finance. According to calculations by the green finance team at the Renmin University of China, green financing requirements in China may reach up to 30 trillion yuan from 2014 to 2020 if environmental factors remain stable. Additionally, the highest green funding needs from 2014 to 2030 are expected to be 123 trillion yuan. Given the substantial demand for funding, fiscal resources alone will not suffice. The implementation of green finance is necessary to attract more financial resources towards sustainable economic growth and transformation. Effective financial and market systems enable limited fiscal funds to attract social capital several times over into green industries, creating new growth opportunities and expediting the green transformation of economic structures.

Therefore, exploring the ecological impact of financial development and technological innovation is important for balancing economic growth and environmental degradation while promoting high-quality economic development in China. This paper focuses on Chinese provincial panel data from 2005 to 2020, using a dynamic spatial STIRPAT panel model to examine the effects of financial development and technological innovation on environmental pollution.

The main research of the paper shows the following: (1) Financial development indirectly exacerbates environmental pollution in China. The development of the financial industry reduces production costs for companies, leading to increased energy consumption and pollution emissions and resulting in further environmental degradation. (2) Technological innovation can effectively reduce local environmental pollution. The advancement of pollution treatment and cleaner production technologies promotes the development of technological innovation capabilities and efficiently controls pollutant discharge. (3) Regions with high pollution levels are heavily concentrated, and China’s environmental pollution exhibits a strong positive spatial autocorrelation.

The remaining structure is arranged as follows: Section 2 shows the literature review; Section 3 describes the theoretical models, empirical models, variables, and data sources; Section 4 shows the results about the spatial STIRPAT; Section 5 presents the main conclusions and policy implications.

## 2. Literature Review

Regarding environmental pollution research, many scholars have carried out a great deal of work from different perspectives such as economic growth, foreign direct investment (FDI), government regulation, and industrial structure. For example, Grossman and Krueger (1991) [8] studied the relationship between sulfur dioxide and dust emissions and per capita income and pointed out that there was an inverted U-shaped pattern between economic growth and environmental pollution, and they defined this pattern as the environmental Kuznets curve (EKC).

According to the EKU curve, there is an inverted U-shaped relationship between pollution and per capita income, which implies that economic development exacerbates local environmental pollution at the early stage, while economic development tends to improve the local environment after the per capita income reaches a certain inflection point. In the following two decades, a large number of studies have been conducted to investigate the relationship between economic growth and environmental pollution from both theoretical and empirical perspectives. The differences in the theoretical models are mainly due to the different mechanisms to achieve the “inverted U” shape shift in environmental pollution. For example, the models of John and Pecchenino (1994) [9] and Selden and Song (1995) [10] emphasized that an “inverted U-shaped” shift in environmental pollution is possible as long as capital is optimally allocated between environmental management and consumption. Lopez and Mitra (2000) [11] demonstrated the existence of an environmental Kuznets curve through a bargaining model between the government and the private sector. Liang and Yang (2019) [12] studied the relationship between economic growth and environmental pollution by using China’s provincial panel data from 2006 to 2015 as a sample, and the results showed that there was a significant inverted U-shaped relationship between the two.

In the area of empirical studies of economic growth and environmental pollution, pioneering work has been conducted by Grossman and Krueger (1995) [13]. In both studies, the authors assumed that pollution is a cubic term function of GDP per capita and regressed the form of this function on cross-country panel data. The regression results showed that the environmental quality does not continue to decline with income and that most air and water pollutants follow an “inverted U-shaped” or “N-shaped” curve with per capita income. Following this, many empirical studies have appeared in the literature, essentially exploring the relationship between economic growth and environmental pollution in the same way and framework. However, the study by Shafik (1994) [14] showed that the relationship between environmental pollution and economic development was not an inverted U-shaped relationship but a monotonically increasing linear relationship while the empirical evidence estimated by Zhao et al. (2019) [15] showed that the GRP per capita between China and the main environmental pollution types were in an “inverted N” shape. Poon et al. (2006) [16] conducted an empirical study on the relationship between China’s environmental pollution and the level of economic development and showed that the two have a more obvious cubic relationship. Nasrollahi et al. (2020) [17] estimated the relationship between population, industrialization, affluence, technology, and sustainability in the Middle East and North Africa and OECD countries from 1975 to 2015, based on the STIRPAT model. The results showed that the slightly negative impact of economic growth on environmental pollution was more significant, but it is negatively affected by population and industrialization. In the verification study of the EKC, some scholars also focused on the relationship between FDI and environmental pollution.

Enterprises in developed countries face higher environmental governance costs, while developing countries adopt relatively lenient environmental protection measures in order to attract foreign investment. Through international investment, transnational corporations transfer high-polluting industries and production chains to developing countries, thus adversely affecting the environment where they invest [18,19]. At present, most relevant studies are based on the “pollution paradise” hypothesis to test the relationship between FDI and environmental pollution. Due to differences in objects, methods, and data, the results are not consistent. One believes that in the early stage of economic development, in order to attract foreign capital inflows, developing countries tend to relax environmental regulatory standards, accelerate the exploitation of natural resources, and produce more pollution-intensive products, thus becoming the “pollution paradise” of developed countries [20,21]. Another view is that compared with local enterprises, foreign enterprises tend to implement uniform environmental standards and adopt cleaner and more advanced production methods, which is conducive to reducing pollution emissions [22]. From the perspective of environmental protection technology spillover, Letchumanan and Kodama (2000) [23] believed that multinational corporations can improve the environment of host countries through a demonstration effect on enterprises in host countries. Pao and Tsai (2011) [24] used the data of the BRIC countries from 1980 to 2007 and found that FDI can promote the carbon emissions of the host country. Based on the panel data of 110 developed and developing countries from 1985 to 2006, the results of Shahbaz et al. (2013) [25] showed a positive correlation between per capita FDI and per capita carbon emissions. Shahbaz and Nasir (2018) [26] analyzed the decisive factors of French carbon emissions from 2004 to 2011, and the results showed that financial development reduced carbon emissions, thereby improving the environmental quality of France.

Environmental regulation, as the core means of national machinery to control environmental pollution, helps to consolidate the path of regional green development and make up for the lack of market governance [27]. For a long time, governments have reduced environmental pollution mainly through administrative control measures such as limiting the amount of pollution discharged, setting discharge standards, and taxing pollution behaviors. From the macro level, the effectiveness of environmental control in reducing environmental pollution mainly depends on the existing institutional framework [28]. By raising the entry threshold of polluting industries and levying a high environmental pollution tax and other regulatory policies, the government forces enterprises to conduct necessary pollution pretreatment and reduce waste emissions. However, the energy efficiency of environmental regulation on environmental pollution treatment depends on the intensity of environmental regulation [29]. From the perspective of optimal contract design, Li et al. (2022) [30] analyzed the environmental regulation of local governments and their fluctuations and believed that maintaining the independence of local government environmental regulation departments can effectively play the role of government environmental regulation.

With the development of the current financial industry and the popularization of the concept of green finance, finance, as an important factor in promoting economic growth and technological innovation, has gradually attracted the attention of scholars at home and abroad for its ecological effects. For example, Jalil and Feridun (2011) [31] analyzed the relationship between financial development and environmental pollution, and the results showed that there was a negative correlation between financial development and carbon dioxide emissions. Shahbaz (2013) [32] studied the relationship between Pakistan’s financial development and environmental pollution and found that the instability of financial development would cause environmental pollution to worsen. Financial development will reduce environmental pollution. Tamazian et al. (2009) [33] believed that financial liberalization and openness are necessary factors to reduce CO_2_ and other polluting gases. Kim et al. (2020) [34] demonstrated that there is a “structural curse” in financial development, where a market-led financial system ultimately exacerbates emissions of gases such as CO_2_. Jalil and Feridun’s (2011) [31] research showed that financial development can significantly reduce environmental pollution. Amri (2018) [35] studied the relationship between financial development and environmental pollution based on the panel data of Tunisia in China from 1975 to 2014 and found that there was a negative correlation between financial development and environmental pollution. Bello and Abimbola (2010) [36] took Nigeria as an example to study the impact of financial development on environmental pollution, and the results showed that from a long-term perspective, financial development has accelerated environmental degradation to a certain extent. Scholtens (2017) [37] believed that the environment should be paid attention to in the process of financial development. Based on the discussion of the transfer effect of financial development on environmental pollution, he analyzed the economic and technical effects of financial industry development on environmental pressure and pointed out that financial development can reduce environment pollution through technical effects. In summary, there are two completely different views on the impact of financial development on environmental pollution.

In addition to the above factors, some scholars have also conducted research on the impact of technological innovation on environmental pollution. Porter and Claas (1995) [38] used theoretical analysis and case studies for the first time and believed that for polluting companies, appropriate regulatory measures can stimulate their technological innovation, reduce environmental regulatory costs, and improve their international competitiveness. Fischer et al. (2003) [39] analyzed the welfare changes brought about by environmental protection instruments endogenous to technological innovation, and the result showed that technological innovation has a significant positive correlation with environmental pollution. Kemfert (2005) [40] analyzed the relationship between corporate investment in technology research and development and energy efficiency from the perspective of regional differences. Research shows that only by strengthening capital expenditures on technology research and development can companies fundamentally reduce environmental pollution governance costs. Based on case analysis, Trianni et al. (2013) [41] pointed out that the improvement of technology will reduce the degree of environmental pollution. Regional innovation can improve the energy efficiency of traditional fossil fuels, thus reducing energy consumption in the production process and achieving the goal of environmental pollution control, which indirectly verifies the inhibition effect of scientific and technological innovation on regional environmental pollution [42]. Technological innovation can minimize the environmental burden of industrial ecological boundaries, which can not only effectively solve the problem of waste disposal and improve the efficiency and sustainability of resource use but also help control greenhouse gas emissions [43]. Grossman et al. (1995) [13] believed that the development, operation, and updating of green technologies can effectively reduce the discharge of waste water, waste gas, and solid waste from enterprises and inhibit regional environmental pollution. In addition, Wang et al. (2019) [44] believed that the emission of pollutants in different industries is heterogeneous. Although technological innovation can improve the energy efficiency of industrial sectors, it will also increase the emission of pollution sources. The current research on the impact of technological innovation on environmental pollution has not reached a consensus.

The existing literature on the impact of financial development and technological innovation on environmental pollution has several shortcomings. These include the following: (1) a lack of exploration of the common relationship between financial development, technological innovation, and environmental pollution, as most research focuses solely on analyzing the impact of either financial development or technological innovation; (2) insufficient theoretical analysis, with most studies only testing the impact empirically; (3) traditional panel regression models mainly used in empirical studies, which may not effectively capture the impact of financial development and the spatial spillover effect of technological innovation on environmental pollution; (4) and lack of a clear conclusion regarding the environmental impact of financial development and technological innovation, emphasizing the need for the latest samples and measurement methods to verify their effects.

Therefore, to address the research gaps mentioned above, this paper presents the following innovations: (1) The first is the construction of a theoretical model to discuss the joint impact of financial development and technological innovation on environmental pollution. The model is based on game theory, which enables a discussion of the mechanism of financial development and technological innovation on environmental pollution from a corporate perspective. (2) The second is the analysis of the impact of financial development and technological innovation on environmental pollution from an empirical perspective using China’s provincial panel data from 2005 to 2020. This analysis is based on the spatial STIRPAT model combined with spatial measurement methods. The dynamic spatial panel model is used to test the impact of China’s financial development and technological innovation on environmental pollution.

However, there are still areas for improvement in this article. Due to data availability, the empirical test data used are provincial panel data, and the regional level of cities is not considered. China’s urban environmental pollution is more severe, and cross-border pollution control between cities is more effective and easier to use for coordinating implementation than between provinces.

## 3. Methods

### 3.1. Theoretical Model

The Gounod duopoly model is considered to be one of the most widely used models in industrial organization theory. Its advantage lies in its ability to accurately describe the competitive process and the final equilibrium outcome among oligopolistic firms in an industry by using output as the decision variable. This paper argues that the Gounod model is profoundly thoughtful and can be used as a reasonable abstraction for the industry of monopolistic competitive market dominated by state-owned enterprises. This paper explores the impact of financial development and technological innovation on environmental pollution by constructing a double oligarchy model to reveal the impact mechanism. Assume that there are only two companies in the market, which are in line with the Cournot duopoly game model, namely enterprise 1 and enterprise 2, and the enterprises produce and sell homogeneous products at the same time. The total output is $q=q_{1}+q_{2}$, where $q_{i}$ represents the output level of firm. Assuming that the total financing demand of the enterprise is ***C***, these funds are mainly used in three parts.

The first part of capital $C_{i1}$ is used for product production, and $c_{i}$ represents the unit output cost, expressed by Equation (1):

(1) $$C_{i1}=c_{i}\times q_{i}$$

The second part of the capital $C_{i2}$ is used for technological development and innovation; $a_{i2}$ represents the ratio of the expenditure of technological innovation $C_{i2}$ to the total production cost $C_{i1}$, expressed by Equation (2):

(2) $$C_{i2}=a_{i2}\times C_{i1}$$

The third part of the capital $C_{i3}$ is used to control pollution emissions and reduce pollution; $a_{i1}$ represents the abatement cost per unit of output, which is expressed by Equation (3):

(3) $$C_{i3}=a_{i1}\times q_{i}$$

Let $c_{i}=f\left(a_{i2}\right),0<f\left(a_{i2}\right)<1,f\left(a_{i2}\right)$ be continuous in the domain $a_{i2}\in \left(0,1\right)$ and $\frac{df}{da_{i2}}<0$, which means that with the increase of technology investment, the average output cost is gradually decreasing. At the same time, let $a_{i1}=g\left(a_{i2}\right),g\left(a_{i2}\right)$ be continuous in the domain $a_{i2}\in \left(0,1\right)$ and $\frac{dg}{da_{i2}}<0$, which means that with the increase of technology input, the cost for emission reduction will decrease. This article will use enterprise 1 as the experimental group and enterprise 2 as the reference group. The total profits of enterprise 1 and enterprise 2 are expressed by Equations (4) and (5), respectively:

(4) $$u_{1}\left(q_{1},q_{2}\right)=q_{1}\times p\left(q\right)-C_{1}=q_{1}\times p-C_{11}-C_{12}-C_{13}=q_{1}\times p-a_{11}\times q_{1}-\left(1+a_{12}\right)\times c_{1}\times q_{1}\;=q_{1}\times p-g\left(a_{12}\right)\times q_{1}-\left(1+a_{12}\right)\times f\left(a_{12}\right)\times q_{1}$$

(5) $$u_{2}\left(q_{1},q_{2}\right)=q_{2}\times p\left(q\right)-C_{2}=q_{2}\times p-C_{21}-C_{22}-C_{23}=q_{2}\times p-a_{21}\times q_{2}-\left(1+a_{22}\right)\times c_{2}\times q_{2}$$

In order to simplify the analysis process, let $p=1-\left(q_{1}+q_{2}\right)$. When the marginal revenue of the enterprise is equal to the marginal cost, that is, $\frac{du_{i}}{dq_{i}}=0$, the enterprise’s response function can be obtained.

The reaction function of enterprise 1 is expressed by Equation (6):

(6) $$q_{1}=\frac{1-q_{2}-\gamma_{1}}{2}$$

The reaction function of enterprise 2 is expressed by Equation (7):

(7) $$q_{2}=\frac{1-q_{1}-\gamma_{2}}{2}$$

where $\gamma_{1}=\left(1+a_{12}\right)\times c_{1}+a_{11},\;\mathrm{and}\;\gamma_{2}=\left(1+a_{22}\right)\times c_{2}+a_{21},$ representing the average total cost of enterprise 1 and enterprise 2, respectively. According to the reaction functions of enterprise 1 and enterprise 2, the equilibrium quantities of the Cournot model of each enterprise are solved as shown in Equations (8) and (9):

(8) $$q_{1}^{*}=\frac{1+\gamma_{2}-2\times \gamma_{1}}{3}$$

(9) $$q_{2}^{*}=\frac{1+\gamma_{1}-2\times \gamma_{2}}{3}$$

Then, the equilibrium price of the Cournot model is shown in Equation (10):

(10) $$p^{*}=\frac{1+\gamma_{1}+\gamma_{2}}{3}$$

With the development of finance, enterprise 1 can obtain the output level that the total financing amount C can achieve in the past at a smaller cost b×C, where b is a constant, and 0<b<1. Currently, the total average cost, equilibrium output, and total emission-reduction expenditure of enterprise 1 are shown in Equations (11)–(13):

(11) $$\gamma_{1}^{\prime }=b\times \gamma_{1}$$

(12) $$q_{1}^{*\prime }=\frac{1+\gamma_{2}-2\times \gamma_{1}^{\prime }}{3}$$

(13) $$C_{13}^{\prime }=a_{11}\times q_{1}^{\prime }=a_{11}\times \frac{1+\gamma_{2}-2\times \gamma_{1}^{\prime }}{3}$$

Combining Equations (8) and (12), we can see that $q_{1}^{*\prime }>q_{1}^{*}$, which indicates financial development, can increase the equilibrium output of enterprises, expand their market share, and strengthen their monopoly power. In addition, $C_{13}^{\prime }>C_{13}$ indicates that financial development will increase the pressure on enterprises to reduce emissions, leading to increased environmental pollution, which is not conducive to the protection of the ecological environment.

Next, we discuss the impact of technological innovation factors on environmental pollution. From the above derivation, using Equations (14)–(16), respectively, expresses the average total cost, equilibrium output, and total emission-reduction expenditure of enterprise 1:

(14) $$\gamma_{1}=\left(1+a_{12}\right)\times f\left(a_{12}\right)+g\left(a_{12}\right)$$

(15) $$q_{1}^{*}=\frac{1+\gamma_{2}-2\times \left[\left(1+a_{12}\right)\times f\left(a_{12}\right)+g\left(a_{12}\right)\right]}{3}$$

(16) $$C_{13}=a_{11}\times q_{1}^{*}=g\left(a_{12}\right)\times \frac{1+\gamma_{2}-2\times \left[\left(1+a_{12}\right)\times f\left(a_{12}\right)+g\left(a_{12}\right)\right]}{3}$$

Respectively, we obtain the derivation of Equations (14)–(16):

(17) $$\frac{d\gamma_{1}}{da_{12}}=f\left(a_{12}\right)+\left(1+a_{12}\right)\times f^{\prime }\left(a_{12}\right)+g^{\prime }\left(a_{12}\right)$$

(18) $$q_{1}^{*\prime }=\frac{dq_{1}^{*}}{da_{12}}=-\frac{2}{3}\times \left[f\left(a_{12}\right)+\left(1+a_{12}\right)\times f^{\prime }\left(a_{12}\right)+g^{\prime }\left(a_{12}\right)\right]$$

(19) $$\frac{dC_{13}}{da_{12}}=g^{\prime }\left(a_{12}\right)\times q_{1}^{*}-g\left(a_{12}\right)\times q_{1}^{*\prime }$$

Because technological innovation is conducive to improving product value and reducing the average total cost of the enterprise by improving the production efficiency of the enterprise, $\frac{d\gamma_{1}}{da_{12}}<0$. Then, from Equations (18) and (19), $q_{1}^{*\prime }=\frac{dq_{1}^{*}}{da_{12}}>0,\frac{dC_{13}}{da_{12}}<0$ means that the greater the R&D investment, the more balanced the output; the higher the output, the lower the abatement cost.

### 3.2. Empirical Model

This paper aims to empirically test the theoretical conclusions presented above by investigating the relationship between technological innovation and pollution control. Specifically, we sought to determine whether there exists a phenomenon whereby increased input leads to higher output, lower emission-reduction costs, and greater balance. To achieve this, we employed a flexible STIPART model, which allows for factor decomposition and the incorporation of relevant variables. Our study investigates the impact of financial development and technological innovation on environmental pollution, and as such, we introduced financial development variables into the STIPART basic model. We used several indicators to represent different aspects of the problem, such as environmental pressure, population size, degree of affluence (measured by level of economic development and per capita GDP), and technological factors. To account for the dynamic persistence of the explained variables, we built a dynamic panel model by introducing a spatial weight matrix and lag term.

Referring to Lim et al. (2019) [45] and Lv et al. (2019) [46], it is necessary to construct a model that allows the flexibility to select other factors to be added according to the research object under the consideration of the original macro conditions, while micro-influencing factors need to be identified when adding them to the model to avoid repeated explanations. According to the basic theory set by the theoretical model, this article discusses the basic conclusions of the theoretical model from the use of the STIRPAT model, including wealth and technological innovation. The STIRPT model is an extension of the IPAT model proposed by Ehrlich et al. (1971) [47] to assess the extent to which population, affluence, and technological factors affect the environment. Since the STIRPT model also contains other variables, this article treats them as control variables, focusing on the financial development level representing wealth variables and technological innovation level. The basic STIRPAT model proposed by Dietz (1997) [48] is widely used in the study of environmental pollution influencing factors, and its structure is shown in Equation (20):

(20) $$I=\alpha \times P^{b}\times A^{c}\times T^{d}e$$

where α is a constant, I represents environmental pressure, P represents population size, A represents economic development level, and A represents technological innovation. b, c, d are their indices, and e is a random error term.

In addition to the above factors affecting environmental pollution, finance has an impact on the ecological environment in the process of promoting economic growth. On the one hand, the development of the financial industry can optimize resource allocation, reduce financing costs, expand production scale, and increase energy consumption and pollutant emissions. On the other hand, adequate financial support can significantly improve the entire market’s technological level and innovation capabilities and indirectly alleviate environmental pressures by promoting the development of green production technologies.

**Figure 1:**
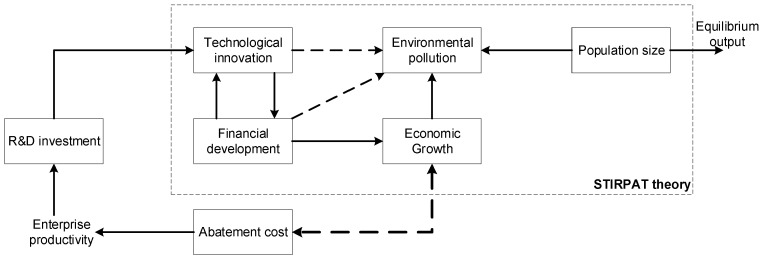
The impact of financial development and technological innovation on environmental pollution.

Based on the STIRPAT model and financial development theory, we can obtain the impact of financial development and technological innovation on environmental pollution, as shown in Figure 1:

According to the theoretical analysis and the influence mechanism in Figure 1, the financial development variables are introduced into the STIRPAT model to obtain the benchmark empirical model set in this article:

(21) $$I=\alpha \times P^{b}\times A^{c}\times T^{d}\times FIN^{\theta }\times e$$

where FIN is the level of financial development, θ is the index corresponding to financial development, and other variables are the same as above.

The paper employs logarithmic linearization to Equation (21) and introduces a spatial weight matrix and hysteresis term to consider the dynamic and spatial effects of environmental pollution. Additionally, the study includes a square term of economic growth to test the environmental Kuznets curve effect, resulting in the dynamic spatial STIRPAT panel model represented by Equation (22).

(22) $$lnENV_{it}=\tau lnENV_{i,t-1}+\rho WlnENV_{it}+\beta_{1}lnFIN_{it}+\beta_{2}lnPAT_{it}+\beta_{3}lnGDP_{it}+\beta_{4}lnPEO_{it}+\beta_{5}lnGDP_{it}^{2}\;+W\left(\delta_{1}lnFIN_{it}+\delta_{2}lnPAT_{it}+\delta_{3}lnGDP_{it}+\delta_{4}lnPEO_{it}+\delta_{5}lnGDP_{it}^{2}\right)+\alpha_{i}+v_{t}+\varepsilon_{it}$$

where τ is the lag term coefficient, $\rho WlnENV_{it}$ represents the spatial influence of the dependent variable, $\alpha_{i}$ is the individual effect, $v_{t}$ is the time effect, and $\varepsilon_{it}$ is the random disturbance term; $lnGDP_{it}^{2}$ is the economic growth square term; β, δ is the estimated coefficient of each main variable, and the other variables are the same as above. W is the spatial weight matrix, which is set as follows: when region i and region j are adjacent, the value of this element is 0. Otherwise, it is 1. To reduce the length, this article mainly reports core and control variables, and the statistical results of the spatial model are all reported in detail.

### 3.3. Data Sources and Descriptive Statistics

The data sample in this article consist of panel data from 30 provinces in China from 2005 to 2020. (Tibet, Taiwan, Hongkong, and Macao are not considered due to missing data.) The selection of each influencing factor and its measurement indicators is explained as follows:

ENV represents the degree of environmental pollution in each province as an explained variable, and the specific construction method of the indicator refers to Li et al. (2022) [30] and Wang et al. (2023) [49]; the data come from the National Bureau of Statistics and the environmental statistical yearbook of each province.

FIN represents the financial development level of each province. The calculation method is the ratio of the total amount of loans issued by local financial institutions to the gross regional product. The data come from the National Bureau of Statistics and the financial statistical yearbooks of each province.

PAT represents the technological innovation strength of each province [5], and the patent application of each province about the quantity is quantified (unit: pieces). The data come from the provincial science and technology statistical yearbooks and the websites of the provincial statistical bureaus.

GDP represents the per capita GDP of each province (deducted and calculated the actual per capita GDP over the years; unit: yuan). The data come from the website of the National Bureau of Statistics.

PEO represents the population size of each province (unit: ten thousand people), including the permanent population in cities and rural areas. The data come from the National Bureau of Statistics and the statistical yearbooks of each province.

The descriptive statistical results of the variables are shown in Table 1:

It can be seen from Table 1 that the average value (mean) of China’s comprehensive environmental pollution index is 0.2123, the minimum value is 0.0007, and the maximum value is 0.7411. The difference between the minimum value and the maximum value is large, indicating that there are large differences in the degree of environmental pollution in different regions; in provinces, the average financial development level is 1.0848 in China, the minimum difference between 0.5372, and the maximum 2.5847 is not much, indicating that the financial development gap between provinces in China is small; the average number of patent applications in each province is 34,964.8934, the minimum is 24, the maximum is 464,118, and the large difference between the minimum maximum values indicates that the technological innovation level of each province is quite different.

## 4. Results

### 4.1. Spatial Correlation Test

**Figure 2:**
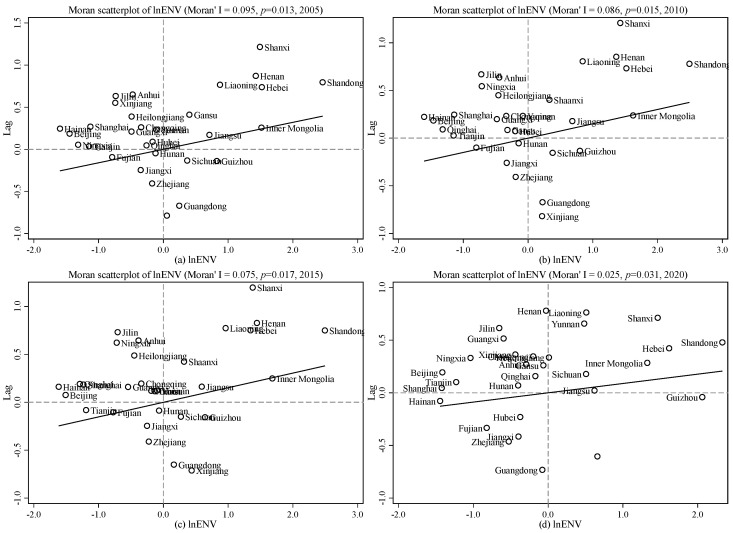
Dynamic changes about spatial autocorrelation (Moran’s I) of environmental pollution. Notes: (**a**–**d**) the dynamic changes about spatial autocorrelation of environmental pollution in 2005, 2010, 2015, and 2020, respectively.

Before performing spatial econometric analysis, a spatial dependence test is required. The Moran scatters plot is a commonly used method for testing spatial dependence. We analyzed the spatial characteristics of environmental pollution in 2005 (Figure 2a), 2010 (Figure 2b), 2015 (Figure 2c), and 2020 (Figure 2d) using Moran scatter plots. The Moran index has been decreasing over the years, all are greater than 0.02, and the *p*-value is less than 0.05, indicating that the Moran’s I index is significant at the 5% level, and the ecological environment pollution of various provinces has a significant positive correlation in China.

**Figure 3:**
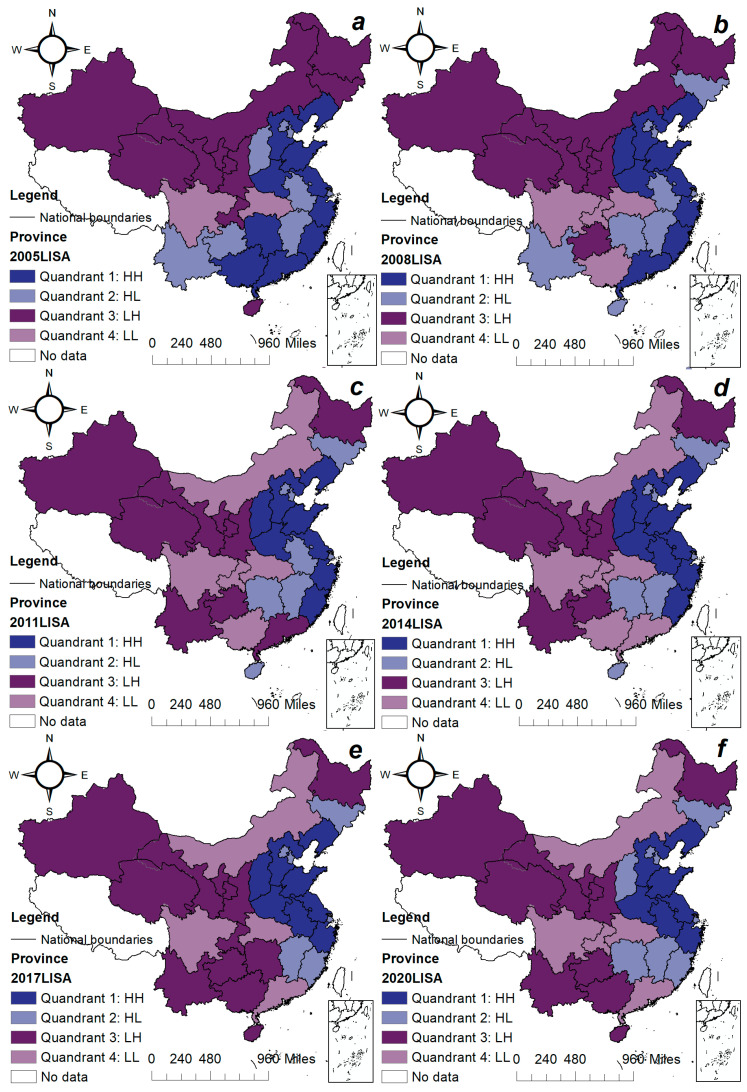
The global Moran scatter displayed spatial clustering in different years. Notes: (**a**–**f**) China’s environmental pollution agglomeration in 2005, 2008, 2011, 2014, 2017 and 2020, respectively.

The Moran scatter chart is divided into four quadrants: The first quadrant indicates that provinces with higher environmental pollution are surrounded by provinces with higher pollution; the second quadrant indicates that provinces with lower environmental pollution are surrounded by provinces with higher pollution. The third quadrant indicates that provinces with lower environmental pollution are surrounded by provinces with lower pollution. The fourth quadrant indicates that provinces with higher environmental pollution are surrounded by provinces with lower pollution. The Moran index values are 0.095, 0.086, 0.075, and 0.025 in Figure 2, respectively, indicating that environmental pollution has significant positive spatial agglomeration characteristics; that is, high-polluted areas are surrounded by high-polluted areas, and low-polluted areas are surrounded by low-polluted areas. In order to show the environmental pollution status of each province more intuitively, Figure 3 shows the provinces corresponding to each of the four scatter diagrams above.

It can be seen from Figure 3 that from 2005 to 2020, China’s overall environmental pollution agglomeration did not change significantly. Provinces where areas with high levels of environmental pollution and surrounding areas with high levels of pollution are clustered, such as Hebei, Liaoning, Jiangsu, Zhejiang, Fujian, Shandong, Henan, etc., indicate that not only are these the regions with more serious ecological environmental pollution, but the surrounding areas are also high in environmental pollution. Guangxi Province has gradually shifted from a high-high environmental pollution agglomeration state to a high-low concentration state, indicating that the ecological environment pollution has not been significantly alleviated in Guangxi Province, but the environmental pollution has begun to decrease in the surrounding areas of Guangxi Province. During the sample period, Yunnan Province shifted from the second quadrant to the third quadrant, indicating that not only is the local environmental pollution relatively low in Yunnan Province, but the environmental quality of the surrounding areas is also improving. While Sichuan Province was in the fourth quadrant during the sample period, the local environmental pollution level is relatively low, and the environmental pressure in the surrounding areas is also relatively small.

It can be seen from Figure 3 that the highest concentration of environmental pollution is in the eastern region of China and the lowest in the western region. The concentration of industrial activity in the eastern coastal areas of China, combined with the relocation of polluting industries from developed countries, has resulted in high environmental pollution across various provinces. Although the western region has absorbed polluting industries from the east, the overall level of environmental pollution remains relatively low due to a less-developed industrial base. Notably, the Guangxi region experienced an improvement in the environmental pollution in 2020, which can be attributed to the initial stages of economic growth. The relationship between economic growth and environmental pollution follows an inverted U-shaped curve, where pollution initially intensifies with economic growth but decreases after the curve reaches its apex.

### 4.2. Benchmark Results

Since China’s environmental pollution has significant spatial spillover effects, it is reasonable to construct a dynamic spatial panel model. The regression results of Equation (22) are shown in Table 2.

The following can be seen from the regression results in Table 2:

(1) *FIN*

This study examines the impact of financial development on local environmental pollution in various provinces of China. Our findings suggest that financial development has a significant and positive effect on local environmental pressure. Specifically, the estimated coefficient of financial development on the local environmental pollution degree is 0.7234, which is statistically significant at the 10% level. This implies that an increase in financial development will lead to a corresponding increase in local environmental pressure. Specifically, a 1% increase in financial development will result in a 0.7234 percentage point increase in local environmental pressure.

Furthermore, we found evidence of spatial spillovers from financial development on surrounding areas’ ecological environment. The estimated spatial spillover coefficient of financial development is −1.5063, which is statistically significant at the 5% level. Our results suggest that local financial development will significantly increase the pressure on the ecological environment in the surrounding areas. One potential explanation for this finding is that the development of the financial industry has made corporate financing more convenient, which may facilitate the expansion of enterprise scale and attract polluting enterprises to the region.

In conclusion, our study provides important insights into the relationship between financial development and environmental pollution in China. Specifically, we found that an increase in financial development leads to a significant increase in local environmental pressure and spillover effects on surrounding areas. Our findings suggest that policymakers should consider the environmental consequences of financial development and implement measures to mitigate its negative impacts on the environment.

(2) *PAT*

This study tests the relationship between technological innovation and local environmental pollution in China. Our findings suggest that technological innovation significantly and negatively affects local environmental pressure. Specifically, the estimated coefficient of influence of technological innovation on local environmental pollution is −0.2432, which is statistically significant at the 1% level. This implies that a 1% increase in technological innovation level leads to a 0.2432 percentage point decrease in local environmental pressure. Our results suggest that the improvement of local technological innovation level can effectively curb local environmental pollution. One potential explanation for this finding is the significant contribution of technological innovation to upgrading the industrial structure, which optimizes the length of the energy supply chain and improves the industrial structure’s advanced and rationalized design.

However, we found no significant evidence of spatial spillovers from technological innovation on surrounding areas’ environmental pollution. Technological innovation’s estimated spatial spillover coefficient is −0.1387, which is not statistically significant at the 10% level. This implies that the improvement of technological innovation in this region does not significantly inhibit the surrounding environmental pollution. One potential explanation for this finding is the imperfect development of the technological market in each province, which prevents the effective diffusion of related technologies between different regions [50,51].

Our study sheds light on the role of technological innovation in curbing local environmental pollution in China. Specifically, we found that increasing technological innovation levels can significantly decrease local environmental pressure, but such improvement may not significantly inhibit surrounding areas’ environmental pollution. Our findings suggest that policymakers should encourage the development of a more robust technological market and facilitate the diffusion of related technologies between different regions to mitigate the negative impact of environmental pollution.

(3) *GDP*

As for *GDP*, this study investigates the impact of economic growth on local environmental pollution in the western region of China. Our findings indicate a significant inverted U-shaped relationship between economic growth and environmental pressure, confirming the environmental Kuznets curve (EKC) hypothesis. Specifically, the estimated primary coefficient of the impact of economic growth on local environmental pollution is 7.3654, and its square coefficient is −0.8685, both of which are statistically significant at the 1% level. This implies that as economic growth increases, local environmental pressure initially rises but eventually decreases after reaching a turning point. This inverted U-shaped relationship is mainly due to the western region’s remote location, which requires the consumption of certain natural resources to achieve economic growth. As a result, enterprises prioritizing economic development over environmental quality have greatly pressured the environment. However, after continuous regional economic development, technical support is provided for environmental improvement, leading to a decline in local environmental pressure.

Moreover, we found that regional economic development also has a significant positive effect on the environmental quality of the surrounding areas. The estimated spatial spillover coefficient of economic growth is −9.9243, which is statistically significant at the 1% level. This suggests that the improvement of regional economic development level is conducive to improving the environmental quality of the surrounding areas.

In conclusion, our study provides insights into the relationship between economic growth and environmental pressure in China’s western region. We found evidence of an inverted U-shaped relationship between economic growth and local environmental pollution as well as significant positive spillover effects of economic growth on the surrounding areas’ environmental quality. Our results suggest that policymakers should balance economic growth and environmental protection to achieve sustainable development in China’s western region.

(4)
PEO

The present study investigates the impact of population size on local environmental pollution in China. Results reveal that the impact coefficient of population size on local environmental pollution is 1.2914, which is significant at the 1% level. This finding suggests that for every one percentage point increase in population size, environmental pressure increases by 1.2914 percentage points, indicating that the expansion of local population size significantly raises local environmental pressure.

Moreover, the spatial spillover coefficient of population size is −0.8129, which is significant at the 1% level. This result indicates that an increase in population size can effectively improve the environmental quality of surrounding areas. In line with national talent policies, attracting high-tech talents with high talent quality to increase the size of the regional population stimulates the innovative vitality of enterprises and, in turn, boosts the development of resource-saving economies.

Furthermore, the study found that the lagging coefficient of environmental pollution is 0.7710, which is significant at the 5% level, suggesting a relatively obvious lag effect in environmental pollution across various regions of China. The degree of environmental pollution in the early stage significantly impacts the environmental pressure in the later stage.

Finally, the study revealed that the value of the spatial autocorrelation coefficient of environmental pollution is 0.1113, which is significant at the 10% level, indicating that China’s environmental pollution presents a relatively obvious spatial agglomeration feature. High (low) pollution areas are often adjacent to high (low) pollution areas, which is consistent with the Moran index test conducted above.

In conclusion, the results of this study suggest that population size significantly impacts environmental pollution in China and that policies aimed at attracting high-tech talents to increase population size may have positive implications for both the environment and the economy. Additionally, the study highlights the importance of addressing the lag effect of environmental pollution and the spatial agglomeration feature of pollution in China.

### 4.3. Robustness Test

In order to test the robustness of the empirical results, this paper analyzes the robustness from two aspects: the construction of the spatial weight matrix and the selection of variables.

#### 4.3.1. The Robustness Test of the Replacement Spatial Weight Matrix

Considering that the weight matrix in the spatial and geographic sense is used above, the spatial weight matrix was reset. We considered the spatial weight matrix in the economic sense, selected the reciprocal of the mean value of the GDP of each region during the sample period as the elements of the matrix, and estimated the above spatial model. The results are shown in Table 3. In spatial econometric modeling, the spatial weight matrix is crucial to capture spatial dependencies among observations. To replace the matrix, a new one must be chosen based on the research question and data characteristics. After re-estimation, heterogeneous results may occur due to differences in the spatial relationships captured by the old and new matrices in this paper as well as variations in matrix size and shape. Understanding such differences is essential for accurate modeling.

According to the regression results in Table 3, the spatial impact coefficient of financial development and technological innovation on environmental pollution has not changed significantly, and the sign of the coefficient is consistent with the previous results. Therefore, the model passes the robustness test.

#### 4.3.2. The Robustness Test of Variable Selection

In order to test that the impact of financial development and technological innovation on environmental pollution is not disturbed by the control variables, we deleted the population scale variable in the model and reconstructed the spatial measurement model for analysis. The results are shown in Table 4.

According to the regression results in Table 4, the spatial impact of financial development and technological innovation on environmental pollution is basically consistent with the conclusions of the original model. The signs of the variable parameters and the spatial autocorrelation coefficient are roughly the same, so the model passes the robustness test.

## 5. Conclusions and Policy Implications

This paper aimed to explore the joint impact of financial development and technological innovation on environmental pollution using game analysis methods. Our findings revealed that while financial development leads to increased corporate production profits, it indirectly exacerbates environmental pollution. On the other hand, technological innovation not only increases the scale of output but also enhances the efficiency of corporate resource utilization, thereby reducing corporate pollution emissions. Based on the results of the game analysis, we conducted an empirical study using panel data from 30 provinces in China spanning from 2005 to 2020. We established a dynamic spatial STIRPAT panel model to examine the spatial impact of financial development and technological innovation on environmental pollution.

After conducting the study, the following findings were obtained: (1) China’s environmental pollution exhibits a strong positive spatial autocorrelation, and regions with high pollution levels are heavily concentrated. (2) The study indicates that an increase in the level of financial development significantly exacerbates local environmental pollution. This is mainly due to the financial industry’s development, which reduces production costs for companies, resulting in an increase in the scale of production, energy consumption, and pollution emissions, leading to further environmental degradation. However, the development of the local financial industry also attracts companies from neighboring areas, which can improve the environmental quality of the surrounding regions. (3) The study suggests that an improvement in technological innovation can effectively reduce the level of local environmental pollution. This is mainly due to the advancement of pollution treatment and cleaner production technologies, which promote the development of technological innovation capabilities and efficiently control pollutant discharge. While the environmental quality of neighboring regions is trending upwards, the effect is not significant, possibly because the local technology does not exert noticeable spillover effects.

Based on the research findings, this paper proposes several policy recommendations to promote a harmonious relationship between economic development and environmental protection in China. Firstly, policymakers should consider the spatial distribution of pollution and develop regional coordination measures to manage environmental pollution. This includes strengthening regional technology exchanges, implementing major environmental control projects, and formulating environmental pollution control measures from a regional perspective. Secondly, a green financial system should be developed to curb environmental pollution by providing financial support to energy-saving and environmentally friendly enterprises. This can be achieved by promoting green bonds, insurance, credit, and other financial instruments. Thirdly, a market-oriented green technology innovation system should be established to increase investment in clean and environmentally friendly technologies, support new environmental protection industries, and accelerate the green transformation of manufacturing. Finally, policymakers should prioritize introducing and applying resource-saving and environmentally friendly technologies, products, and services while attracting foreign direct investment and advanced technologies and funds from developed countries and regions. The promotion of the concept of green environmental protection should also be emphasized.

## Figures and Tables

**Table 1:** Variable descriptive statistics.

Name	Obs	Mean	Standard Deviation	Minimum	Maximum
*ENV*	480	0.2123	0.1536	0.0007	0.7411
*FIN*	480	1.0848	0.3831	0.5372	2.5847
*PAT*	480	34,964.8934	5927.8230	24.0000	464,118.0000
*GDP*	480	33,386.8000	20,691.5100	5052.0000	105,231.0000
*PEO*	480	4533.5660	2610.4540	543.0000	10,724.0000

**Table 2:** Regression results of dynamic spatial panel model.

Variable	Local Area Influence	Surrounding Area Influence
lnFIN	0.7234 * (0.5180)	−1.5063 ** (0.6275)
lnPAT	−0.2432 *** (0.1369)	−0.1387 (0.0534)
lnGDP	7.3645 *** (2.1053)	−9.9243 *** (3.3233)
lnPEO	1.2914 *** (0.2210)	−0.8129 *** (0.3210)
$lnGDP^{2}$	−0.8685 *** (0.1288)	0.6285 *** (0.2241)
ρ	0.1113 * (0.0956)	
τ	0.7710 ** (0.0957)	

Note: The standard error is below the coefficient in the table; *, **, and *** represent that the coefficient is significant at 10%, 5%, and 1%, respectively.

**Table 3:** Model estimation results of replacement space weight matrix.

Variable	Local Area Influence	Surrounding Area Influence
lnFIN	0.9879 * (0.7852)	−2.3201 * (1.9875)
lnPAT	−0.5437 * (0.5437)	0.5980 *** (0.0741)
lnGDP	6.3574 *** (2.7531)	−9.3675 ** (5.9521)
lnPEO	1.8520 (1.0335)	−1.3574 (3.3101)
$lnGDP^{2}$	−1.8574 *** (1.7561)	0.6741 ** (0.3320)
ρ	0.0915 * (0.0752)	
τ	0.3312 * (0.1397)	

Note: The standard error is below the coefficient in the table; *, **, and *** represent that the coefficient is significant at 10%, 5%, and 1%, respectively.

**Table 4:** Model estimation results without population variables.

Variable	Local Area Influence	Surrounding Area Influence
lnFIN	2.0037 * (1.3874)	−2.1001 ** (2.9867)
lnPAT	−1.3420 ** (0.5714)	0.1276 (1.8750)
lnGDP	7.0135 *** (3.1102)	−8.3975 ** (5.8842)
$lnGDP^{2}$	−1.8574 *** (1.7561)	0.6741 ** (0.3320)
ρ	0.2333 * (0.1752)	
τ	0.5984 * (0.1327)	

Note: The standard error is below the coefficient in the table; *, **, and *** represent that the coefficient is significant at 10%, 5%, and 1%, respectively.

## Data Availability

The data presented in this study are available on request from the corresponding author.
